# The Two-Echelon Unmanned Ground Vehicle Routing Problem: Extreme-Weather Goods Distribution as a Case Study

**DOI:** 10.3390/biomimetics10050255

**Published:** 2025-04-22

**Authors:** Chuncheng Fang, Yanguang Cai, Yanlin Wu

**Affiliations:** 1 School of Automation, Guangdong University of Technology, No. 100, West Ring Road, Guangzhou Higher Education Mega Center, Xiaoguwei Street, Panyu District, Guangzhou 510006, China; fcc_168@163.com (C.F.); ylwucomm@foxmail.com (Y.W.); 2 Department of Mechanical and Electrical Engineering, Jieyang Polytechnic, Jieyang 522051, China; 3 School of Artificial Intelligence, Guangzhou Institute of Science and Technology, Guangzhou 510540, China

**Keywords:** two-echelon unmanned ground vehicle routing problem, hybrid artificial bee colony–wild horse optimizer, goods distribution, extreme weather

## Abstract

In extreme weather conditions, the use of unmanned ground vehicles (UGVs) for material distribution enhances safety. We introduce a two-echelon unmanned ground vehicle routing problem (2E-UGVRP) and proposes a hybrid Artificial Bee Colony–Wild Horse Optimizer (HABC-WHO) algorithm to solve it. In this approach, the optimal solution obtained from the Artificial Bee Colony algorithm replaces the worst solution of the Wild Horse Optimizer. To further improve the algorithm’s performance, strategies such as large neighborhood search, two-optimization (2-Opt) operation, and satellite subpath crossover are incorporated. The algorithm’s effectiveness is demonstrated through the solution of 43 benchmark instances, with performance comparisons against a Genetic Algorithm (GA), Discrete Wild Horse Optimizer (DWHO), and Discrete Artificial Bee Colony–Fixed Neighborhood Search (DABC-FNS). The results clearly show the significant superiority of the proposed algorithm. Additionally, the algorithm is applied to material distribution by two-echelon UGVs under extreme weather conditions, yielding promising results. Experimental findings indicate that the algorithm exhibits strong solving capability and high precision.

## 1. Introduction

With the continuous growth of e-commerce, urbanization leads to increased transportation requirements in cities, and the last-mile delivery (LMD) is the most expensive part of the supply chain, as high personnel costs incur [1]. Labor cost accounts for the majority of logistics costs. Thus, transportation companies do their best efforts to find a solution for cutting down dispatch costs. Then, the application of unmanned vehicles in logistics can efficiently cut down the dispatch costs and improve the efficiency of transportation.

Recently, several unmanned vehicles have emerged. Starship Technologies, which is an Estonian start-up company, develops autonomous robots for last-mile deliveries. Starship’s robots move along sidewalks and weigh no more than 40 pounds while fully loaded [2]. In 2016, Cainiao launched its first last-mile delivery robot, Xiao G. [3], and Domino’s unveiled its next-generation delivery robot, named DRU (Domino’s Robotic Unit). Dispatch launched its first autonomous delivery vehicle, Carry, which was designed to operate exclusively on bicycle lanes and sidewalks [3]. Piaggio Fast Forward (PFF) positions its products as ‘optimally sized transport solutions’, bridging the gap between drones and automobiles. One of its commercially developed models is Gita [3]. They can be applied to deliver parcels or groceries directly from warehouses or specialized hubs. After robots have finished their dispatch mission, they can autonomously return to their warehouse or hub. For safety, the robots are only permitted to move at pedestrian speed. For reducing the costs and the CO_2_ emissions, improving consumer satisfaction, and building an environmentally friendly economy, an unmanned ground vehicle is an excellent choice for logistics. COVID-19, the global pandemic, has made us realize the importance of contactless delivery services and robotic automation, which will further promote the widespread application of unmanned delivery technology.

Currently, there is extensive literature on parcel delivery using trucks and drones, while studies focusing solely on parcel delivery with unmanned ground vehicles (UGVs) are relatively scarce. The vehicle routing problem (VRP) is an NP-hard optimization problem that aims to determine a set of least-cost delivery routes from a depot to a set of geographically scattered customers, subject to side constraints [4].

**Figure 1:**
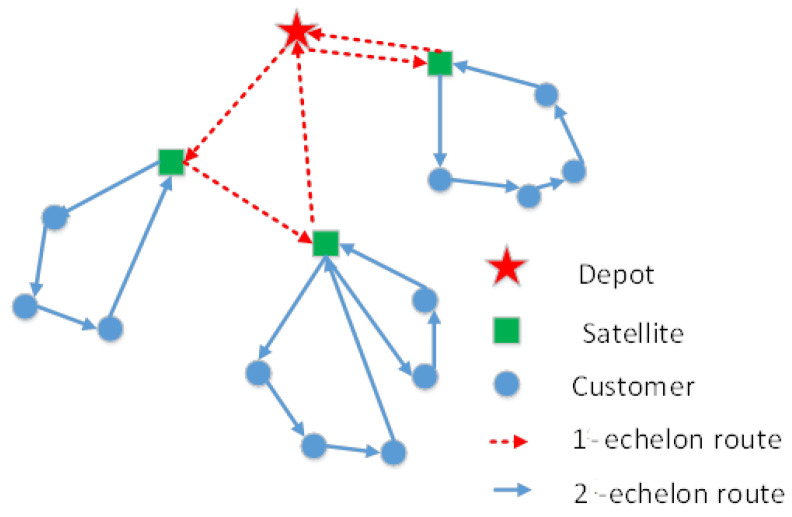
Two-echelon distribution model.

In enclosed areas such as residential communities and campuses, UGVs can be used to reduce delivery costs and carbon emissions. To alleviate traffic congestion and enhance delivery efficiency, the concept of two-echelon delivery has been introduced [5]. The 2E-VRP is a variant of the VRP, involving a two-echelon distribution network with a central depot, a set of satellites, and a group of end customers. In the first echelon, the delivery task is typically handled by larger-capacity vehicles, while the second echelon involves smaller-capacity vehicles. Goods are first transported from the depot to the satellites by the first-echelon vehicles, then delivered to the end customers by second-echelon vehicles. Each customer is served by exactly one second-echelon vehicle. The objective is to minimize the total routing cost of both echelons. Figure 1 illustrates an example of the 2E-VRP.

Our research focuses on the practical application of two-echelon logistics distribution using UGVs under extreme weather conditions, which is a variant of the 2E-VRP. In this model, the first stage involves large-capacity UGVs delivering goods, while the second stage involves smaller-capacity UGVs for final deliveries. Due to the unsuitability of drones in extreme weather and to prevent risks to human life during the delivery process, UGVs are a more appropriate choice for such conditions. We refer to this model as the two-echelon unmanned ground vehicle routing problem (2E-UGVRP). The contribution and innovation of this paper is the development of a hybrid Artificial Bee Colony–Wild Horse Optimizer (HABC-WHO) to solve the problem. The Wild Horse Optimizer (WHO) [6], as a recently proposed metaheuristic, demonstrates strong local search capabilities when integrated with search strategies, while the Artificial Bee Colony (ABC) algorithm excels in global exploration. By replacing the worst solution in the WHO with the optimal solution from the ABC algorithm, the proposed hybrid algorithm enhances the WHO’s global search ability, mitigates the risk of premature convergence, and improves overall solution quality. Some algorithms are primarily suitable for small-scale problem instances and demonstrate limited effectiveness when tackling large-scale cases, such as linear programming methods. Certain algorithms, like Genetic Algorithms (GAs), are susceptible to premature convergence, while others, such as Particle Swarm Optimization (PSO), often exhibit slow convergence. The algorithm developed in this study effectively mitigates these shortcomings by accelerating convergence while maintaining solution diversity, thereby avoiding premature convergence and enhancing solution accuracy.

The remainder of the paper is organized as follows. Section 2 provides a non-exhaustive literature review. Section 3 presents a detailed problem description. Section 4 provides the mathematical formulation for the 2E-UGVRP. Section 5 explains the solution methodology developed to solve the problem. Section 6 presents extensive computational results. Section 6 and Section 7 present the computational tests and the application to real-world problems, respectively. Finally, Section 8 summarizes the work and highlights future research challenges.

## 2. Literature Review

The concept of the two-echelon vehicle routing problem (2E-VRP) was first introduced by Jacobsen and Madsen [5] in 1980 through a newspaper distribution system. Crainic, Ricciardi, and Storchi [7] later proposed the concept of a two-echelon delivery network for urban freight distribution, characterized by two levels of platforms. Gonzalez-Feliu et al. [8] were the first to introduce the two-echelon capacitated vehicle routing problem (2E-CVRP). Crainic, Ricciardi, and Storchi [9] refined the definition of the 2E-CVRP, describing it as a two-layer, time-dependent, synchronized, multi-route, multi-warehouse, multi-product, heterogeneous fleet problem with both hard (satellite) and soft (customer) time windows at each echelon. Sluijk et al. [10] explored the two-stage capacitated vehicle routing problem in urban logistics. Perboli, Tadei, and Vigo [11] introduced a mathematical model for the 2E-VRP with a single warehouse, naming it the 2E-VRP. In this problem, vehicles in the first stage transport goods from a warehouse to a satellite located at the echelon boundary. At the satellite, goods are unloaded from the first-stage vehicles and loaded onto smaller second-stage vehicles that meet the delivery requirements. Subsequently, the second-stage vehicles perform deliveries from the satellite to customers. Crainic et al. [12] designed a multi-heuristic algorithm that iteratively solved subpath problems in both stages while adjusting the satellite workload connecting them. In multi-echelon vehicle routing problems, deliveries from the warehouse to the customer are managed by rerouting and consolidating goods through intermediate satellites or distribution centers (cross-docks or distribution centers) [13].

Murray and Chu [14] were the first to study the truck–drone delivery problem, flying sidekick traveling salesman problem (FSTSP), where trucks serve as first-echelon delivery vehicles and drones serve as second-echelon delivery vehicles. Drones are launched from the truck at specified locations to deliver goods to customers. Agatz et al. [15] noted that trucks typically have longer ranges and can carry many parcels, while drones offer faster, cheaper, and more flexible deliveries. However, drone delivery is limited by payload and flight distance, which can be mitigated through the truck–drone delivery problem, improving delivery efficiency. When multiple trucks and drones are involved, the problem is referred to as the vehicle routing problem with drones (VRPD) [14]. Zhou et al. [16] proposed a new variant of the VRPD, where drones performed multiple back-and-forth trips while the paired vehicle stopped at customer nodes. Li et al. [17] introduced a model for homogeneous fleets of trucks and drones used to deliver parcels. In this model, each truck carries the same number of drones, and the characteristics of each truck and drone are identical. The problem is solved using the adaptive large neighborhood search (ALNS) heuristic algorithm.

The literature [18] also explores variants of the 2E-VRP, including the two-echelon stochastic vehicle routing problem with loading bay reservation strategy, which addresses parking and loading inefficiencies and is solved using a hybrid genetic algorithm. Zhong et al. [19] proposed an Artificial Bee Colony Genetic Algorithm (ABCGA) to solve standard 2E-VRPs with up to 51 customers. References [19,20,21,22] investigate the two-echelon vehicle routing problem for electric vehicles (2E-EVRP). Additionally, the two-echelon electric vehicle routing problem (2E-EVRP) has been studied, where charging or battery-swapping stations expand the transportation network. Crainic [23] and Marques [24] addressed the use of satellites as automatic charging stations, with secondary deliveries considered as multiple trips in the 2E-VRP. Vincent et al. [25] proposed a new 2EVRP variant incorporating time windows, intermediate facilities, and occasional drivers, termed the two-echelon vehicle routing problem with time windows, intermediate facilities, and occasional drivers (2E-VRPTW-IF-OD). A hybrid adaptive large neighborhood search (HALNS) algorithm was used for solving this variant. Lehmann and Winkenbach [26] developed a hybrid approach integrating exact methods with adaptive large neighborhood search for the time-constrained two-echelon routing problem. Dellaert et al. [27] introduced a variant of the 2E-VRP with customer hard time windows, resulting in synchronization issues between first- and second-echelon vehicles. Zamal et al. [28] developed an adaptive large neighborhood search algorithm to solve the two-echelon vehicle routing problem with pickups and deliveries. Lehmann and Winkenbach [26] designed a solution for the simultaneous pickup and delivery problem in a multi-trip 2E-VRP, while Mhamedi et al. [29] applied a branch-and-bound algorithm to solve a two-stage vehicle routing problem with time windows. They introduced effective deep dual-optimal inequalities and applied known valid inequalities. Dellaert N et al. [30] proposed a two-stage vehicle routing problem with time windows and multiple products. Zhou et al. [31] used a variable neighborhood tabu search algorithm for solving the 2E-VRP with time windows and simultaneous pickup and delivery. Kim et al. [32] explored the two-echelon hetero-collaborative routing problem (2E-HCRP), which facilitated parcel transfers between couriers and customer nodes, optimizing last-mile deliveries. Al Theeb et al. [33] proposed a heuristic solution based on greedy randomized adaptive search procedure to address the multi-objective two-echelon vaccine distribution problem. Grangier et al. [34] studied the two-echelon multiple-trip vehicle routing problem with satellite synchronization (2E-MTVRP-SS), solving it with an adaptive large neighborhood search method. Marques et al. [35] proposed an improved branch-price-and-cut algorithm to solve the two-stage vehicle routing problem with capacity constraints.

Anderluh et al. [36] considered a distribution system involving cargo bikes in the second echelon, with a real-world instance based on Vienna, Austria. Kergosien et al. [37] examined logistics problems at a hospital complex in Tours, France, where the first echelon involved vehicle transport between hospital units, and the second echelon focused on constructing delivery routes within individual buildings.

Currently, two main approaches are used to solve the 2E-VRP: exact algorithms such as Mixed-Integer Linear Programming (MILP) [2,14] and dynamic programming [38], and metaheuristic algorithms, including the Horsefly Algorithm [39], adaptive large neighborhood search (ALNS) [17,32,40], variable neighborhood search [31], tabu search [31,41], Genetic Algorithms [18,19,37], and Artificial Bee Colony (ABC) [19,42]. Currently, no algorithm outperforms all others across all problem instances. Table 1 provides an overview of the main solution methods for 2E-VRP.

The previous literature on two-echelon delivery problems has primarily considered truck–drone or truck–unmanned vehicle combinations. Our research, for the first time, introduces the two-echelon unmanned ground vehicle routing problem (2E-UGVRP), where unmanned ground vehicles (UGVs) are utilized in both delivery echelons. A novel solution approach, the hybrid Artificial Bee Colony–Wild Horse Optimizer (HABC-WHO), is proposed for this problem. This paper addresses a research gap and provides new insights into unmanned logistics optimization.

## 3. Problem Description

The two-echelon unmanned ground distribution network involves two fleets of vehicles that handle transfers at different stages [45]. In the first echelon, unmanned vehicles (first echelon) collect goods from the central depot and distribute them to several intermediate facilities known as satellites, where the goods are unloaded and transferred to another type of unmanned vehicle for further transportation to customers (second echelon). The central depot has a limited number of unmanned vehicles, each capable of servicing multiple satellite facilities. In the first echelon, if the delivery volume to a satellite exceeds the capacity of a single unmanned vehicle, multiple first-echelon vehicles may service that satellite. In the second echelon, since each customer’s demand is less than the capacity of the second-echelon vehicles, each customer is served by exactly one unmanned vehicle.

In the two-echelon unmanned ground vehicle routing problem, we define as arc (*i*,*j*) the direct route connecting node i to node j. If both nodes are satellites or if one is the depot and the other is a satellite, we define the arc as belonging to the first-echelon routing, while if both nodes are customers or if one is a satellite and the other is a customer, the arc belongs to the second-echelon routing. The first echelon connects the depots to the satellites; the second echelon is where the cargo is delivered from the satellites to the customers.

We denote the central depot as $V_{0}$, the satellite facilities as Vs, and the customers as *C*. The first-echelon fleet departs from the central depot, services the satellite facilities, and returns to the depot. The second-echelon fleet departs from the satellite facilities, serves the end customers, and returns to the satellite facilities. The capacity of each vehicle is fixed, with the first-echelon vehicles having a larger capacity than the second-echelon vehicles. The objective is to minimize the total transportation cost while meeting the vehicle capacity constraints and ensuring service to all customers. All customer demands are known, and since each customer’s demand is smaller than the capacity of the second-echelon vehicles, they are served by a single second-echelon vehicle.

In our model, we temporarily do not consider the fixed cost of vehicle transportation, assuming it to be constant. We focus on minimizing the total length of the transportation routes. Therefore, the total transportation cost consists of two components: the cost from the central depot to the satellites and the cost from the satellites to the end customers.

## 4. Mathematical Formulation

Let an undirected graph G=(N,E) be given, where the node set *N* is partitioned as $N=V_{0}\cup V_{S}\cup V_{C}$. $V_{0}=0$ denotes the central depot, $V_{S}=\left\{1,2,\ldots ,n_{s}\right\}$ represents the set of $N_{s}$ satellites, and $N_{C}=\left\{n_{s}+1,n_{s}+2,\ldots ,n_{s}+n_{c}\right\}$ denotes the set of $n_{c}$ customers. The edge set *E* is defined as $E=\left\{\left\{0,j\right\}:j\in N_{S}\right\}\cup \left\{\left\{i,j\right\}:i,j\in N_{S}\cup N_{C},\;i<j\right\}$.

Let $m_{1}$ and $m_{2}$ denote the number of vehicles available in the first and second echelons, respectively. The parameter $M_{sk}$ represents the maximum number of second-echelon delivery routes that can be initiated from satellite *k*. Vehicle capacities in the first and second echelons are represented by $K_{1}$ and $K_{2}$, respectively. The demand of each customer $i\in N_{C}$ is denoted by $d_{i}$, and $c_{ij}$ is the cost associated with traversing arc (i,j)∈E.

Each satellite $k\in N_{S}$ is associated with a per-unit handling cost $F_{k}$, which includes loading and unloading operations. The binary variable $D_{k}$ equals one if satellite *k* is activated in the distribution network, and zero otherwise. The variables $Q_{ij}^{1}$ and $Q_{ij}^{2}$ represent the volume of goods transported through arc (i,j) in the first and second echelons, respectively. Let $x_{ij}\in Z^{+}$ denote the number of unmanned ground vehicles traversing arc (i,j) in the first-echelon network. The binary variable $y_{ij}^{k}\in \left\{0,1\right\}$ equals one if arc (i,j) is included in a second-echelon routing path originating from satellite $k\in N_{S}$, and zero otherwise. The binary variable $z_{kj}\in \left\{0,1\right\}$ equals one if customer $j\in N_{C}$ is assigned to satellite *k*, and zero otherwise.

The model to minimize the total cost of the system may be formulated as follows:

(1) $$min(\sum_{i,j\in V_{0}\cup V_{s}}C_{ij}x_{ij}+\sum_{k\in V_{s}}\sum_{i,j\in V_{s}\cup V_{c},i\neq j}c_{ij}y_{ij}^{k}+\sum_{k\in V_{s}}F_{k}D_{k}),$$

Let $F_{k}D_{k}$ represent the fixed cost for each satellite’s participation in the operation. To simplify the model, we omit the fixed cost, and thus Function (1) can be simplified to (2):

(2) $$min(\sum_{i,j\in V_{0}\cup V_{s}}C_{ij}x_{ij}+\sum_{k\in V_{s}}\sum_{i,j\in V_{s}\cup V_{c},i\neq j}c_{ij}y_{ij}^{k}),$$

subject to

(3) $$\sum_{i\in V_{s}}x_{0}<m_{1},$$

(4) $$\sum_{j\in V_{0}\cup V_{s},j\neq k}x_{jk}=\sum_{j\in V_{0}\cup V_{s},i\neq k}x_{ki},$$

(5) $$\sum_{k\in V_{s}}\sum_{j\in V_{c}}y_{ij}^{k}\leq m_{2},$$

(6) $$\sum_{j\in V_{c}}y_{ij}^{k}\leq m_{sk},\forall k\in V_{s},$$

(7) $$\sum_{j\in V_{c}}y_{kj}^{k}=\sum_{j\in V_{c}}y_{jk}^{k},\forall k\in V_{s},$$

(8) $$\sum_{i\in V_{0}\cup V_{s},i\neq j}Q_{ij}^{1}-\sum_{i\in V_{0}\cup V_{s},i\neq j}Q_{ji}^{1},=\left\{\begin{array}{ccc} \; & D_{j}, & j\;\mathrm{is}\;\mathrm{not}\;\mathrm{the}\;\mathrm{depot},\\ \; & \sum_{i\in V_{C}}-d_{i}, & \mathrm{otherwise},\forall k\in V_{s}\cup V_{C}, \end{array}\right.$$

(9) $$\sum_{i\in V_{0}\cup k,i\neq j}Q_{ijk}^{2}-\sum_{i\in V_{0}\cup k,i\neq j}Q_{jik}^{2},=\left\{\begin{array}{ccc} \; & z_{kj}D_{j}, & j\;\mathrm{is}\;\mathrm{not}\;a\;\mathrm{statellite},\\ \; & -Dj, & otherwise,\forall j\in V_{s}\cup V_{0}, \end{array}\right.$$

(10) $$Q_{ij}^{1}\leq k^{1}x_{ij},\forall i,j\in V_{s}\cup V_{0},i\neq j,$$

(11) $$Q_{ij}^{2}\leq k^{2}y_{ij}^{k},\forall i,j\in V_{s}\cup V_{c},i\neq j,\forall k\in V_{s},$$

(12) $$\sum_{i\in V_{s}}Q_{iv_{0}}^{1}=0,$$

(13) $$\sum_{j\in V_{c}}Q_{jkk}^{2}=0,\forall k\in V_{s},$$

(14) $$y_{ij}^{k}\leq z_{kj},\forall i\in V_{s}\cup V_{c},j\in V_{c},\forall k\in V_{s},$$

(15) $$y_{ji}^{k}\leq z_{kj},\forall k\in V_{s},\forall j\in V_{c},$$

(16) $$\sum_{i\in V_{s}\cup V_{c}}y_{ij}^{k}=z_{kj},\forall k\in V_{s},\forall j\in V_{c},$$

(17) $$\sum_{i\in V_{s}}y_{ji}^{k}=z_{kj},\forall k\in V_{s},\forall j\in V_{c},$$

(18) $$\sum_{i\in V_{s}}z_{kj}=1,\forall j\in V_{c},$$

(19) $$y_{kj}^{k}\leq \sum_{i\in V_{s}\cup V_{0}}x_{kj},\forall k\in V_{s},\forall j\in V_{c},$$

(20) $$y_{kj}^{k}\in \left\{0,1\right\},\forall k\in V_{s}\cup V_{0},\forall i,j\in V_{c},$$

(21) $$z_{kj}\in \left\{0,1\right\},\forall k\in V_{s}\cup V_{0},\forall j\in V_{c},$$

(22) $$x_{kj}\in Z^{+},\forall k,j\in V_{s}\cup V_{c},$$

(23) $$Q_{ij}^{1}\geq 0,\forall i,j\in V_{s}\cup V_{c},$$

(24) $$Q_{ijk}^{2}\geq 0,\forall i,j\in V_{s}\cup V_{c},\forall k\in V_{c},$$

Constraint (3) limits the number of vehicles used by the first-echelon fleet to the total number of vehicles available at the central depot. Constraint (4) specifies that if k $\in V_{0}$, each first-echelon route must begin and end at the depot, meaning the unmanned vehicles in the first-echelon fleet start their route at the central depot and return there upon completing deliveries. Constraint (5) restricts the number of second-echelon unmanned vehicles to the total available in the second-echelon fleet. Constraint (6) imposes capacity restrictions on the satellite depots. Constraint (7) ensures vehicle flow balance at the satellite depots, maintaining consistency in the number of second-echelon vehicles entering and leaving each satellite. Constraint (8) requires that the total volume dispatched from the central depot equals the total demand of all customers. Constraint (9) enforces volume balance at the satellite nodes, ensuring that the inbound volume matches the outbound volume, and prevents the formation of sub-tours. Constraint (10) ensures that the total delivery volume on the first-echelon routes does not exceed the cumulative capacity of all first-echelon unmanned vehicles. Constraint (11) similarly ensures that the total delivery volume on the second-echelon routes does not surpass the total capacity of the second-echelon unmanned vehicles. Constraints (12) and (13) stipulate that routes must be completed before the vehicles return to their respective depots. Constraints (14) and (15) ensure that customer j is assigned to satellite k only if satellite k is responsible for delivering goods to customer j, thereby allocating the customer to the service area of the corresponding satellite. Constraints (16) and (17) ensure that each customer is serviced by exactly one second-echelon unmanned vehicle. Constraint (18) mandates that each customer is assigned to only one satellite. Constraint (19) establishes that a second-echelon unmanned vehicle can only depart from a satellite after the first-echelon vehicle has delivered goods to that satellite. Constraints (20) through (24) allow multiple first-echelon unmanned vehicles to service a single satellite depot if necessary.

## 5. Solution Method

We first assign customers to satellites and propose a hybrid Artificial Bee Colony–Wild Horse Optimizer (HABC-WHO) using K-means clustering to solve the 2E-UGVRP.

### 5.1. Construction of Initial Solutions

The initial solutions are constructed using a clustering approach. Specifically, all customers are grouped using the K-means clustering algorithm, and each customer is assigned to the nearest satellite. The primary paths are then generated through a random permutation to form the initial solution.

### 5.2. The Principle of Wild Horse Optimizer (WHO)

The Wild Horse Optimizer (WHO) [6] is a novel metaheuristic algorithm proposed by Iraj Naruei and Farshid Keynia in 2021, designed to solve optimization problems in continuous systems. The algorithm is inspired by the foraging, mating, and group leadership behaviors observed in wild horses. A wild horse herd consists of stable family groups, each led by a stallion (the leader), along with one or more mares and their offspring. The stallion leads the group members in search of suitable habitats. The basic principles of the Wild Horse Optimizer include the following five components:

(a) Creation of initial population, grouping of herds, and selection of leaders: An initial population is randomly generated and grouped. Each group is led by a stallion, with the remaining members consisting of mares and offspring [6].

(b) Foraging and mating behaviors of wild horses: Foals spend most of their time foraging within the group. In the foraging process, the stallion is considered the center of the foraging area, and group members move and explore around the leader within varying radii. A foal may leave group i and join a temporary group, while another foal may leave group j and join the same temporary group. If these two foals are of opposite sex and not closely related, they can mate. The offspring produced from this mating must leave the temporary group and join another group (e.g., group k), forming the next generation. This process of leaving, mating, and breeding is repeated by all foals within the herd [6].

(c) Leadership of the group by the stallion: The stallion leads its group members to more favorable habitats. If the current group occupies a dominant position in a given area, they continue to stay there. However, if another group holds a more dominant position in the same area, the current group must relocate. The habitat represents the current optimal solution [6].

(d) Communication and selection of leaders: Leaders are selected based on fitness. If a group member has a better fitness value than the current leader, the leader’s position is exchanged with the member. The leader’s position represents the best solution for the group [6].

(e) Saving the optimal solution: The fitness values of the leaders of all groups are compared, and the group leader with the best fitness value represents the global optimal solution. This solution is then stored [6].

### 5.3. Solution Encoding and Decoding

**Figure 2:**
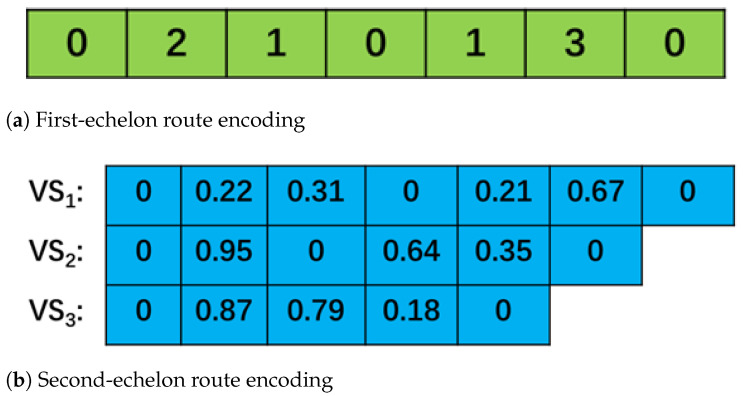
Distribution route scheme encoding.

The paths for the first and second stages are encoded separately. In the first stage, the number of satellites is limited, so we use discrete encoding; in the second stage, we use continuous encoding. Suppose the first stage consists of 3 satellite stations, with the encoding format shown in Figure 2. The second-stage path represents the corresponding customers, which are decoded into integer paths using the Min-position Matched Value (MPMV) method [46]. The first- and second-stage paths are separated by ‘0’, where the numbers before and after ‘0’ represent different vehicles delivering goods. The path in Figure 2a, 0→2→1→0→1→3→0, indicates that two primary unmanned vehicles are involved in the delivery. The first vehicle delivers to satellite stations 2 and 1, while the second vehicle delivers to satellite stations 1 and 3. Figure 2b shows the customer codes corresponding to the satellite deliveries, where the customers belonging to S1 are served by two vehicles.

**Figure 3:**
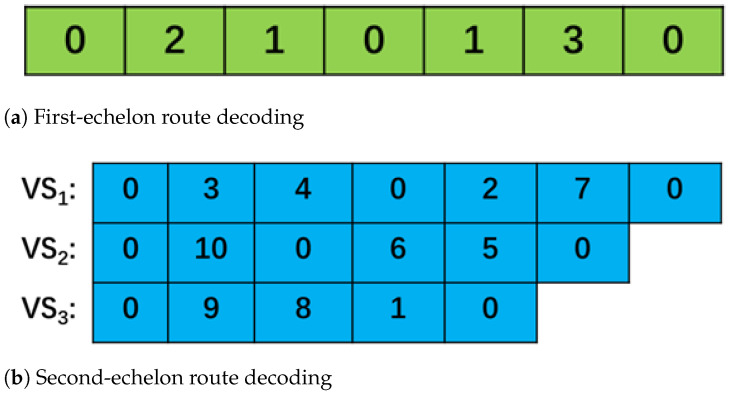
Distribution route scheme decoding.

The WHO is an optimization algorithm designed for continuous problems, and the solution it represents is continuous. Therefore, to solve the 2E-UGVRP, the solution represented by WHO needs to be discretized and decoded to represent the customer delivery path. The decoding method used here is the Min-position Matched Value (MPMV) [46] method. After decoding the scheme in Figure 2 using MPMV, the corresponding delivery scheme is shown in Figure 3.

### 5.4. Wild Horse State Updates

The positions of stallions and foals are defined as $X_{i}=\left(X_{i1},X_{i2},\ldots ,X_{in}\right)$, i∈(1,2,…,popN), where $X_{i}$ represents the *i*th individual, and popN∈(N+) represents the population size; *n* is the number of customers.

The foal grazing behavior is carried out according to (25):

(25) $$X_{G,i}^{j}=2Zcos\left(2\pi RZ\right)\times \left(Stallion^{j}-X_{G,i}^{j}\right)+Stallion^{j},$$

The $Stallion^{j}$ is the leader position, the optimal individual in this group. $X_{i,G}^{j}$ is the current position of the group member. *R* is a random number within [−2, 2], which mainly controls the angle between individuals and the leader. The adaptive mechanism *Z* is calculated according to (26)–(28). The symbol ‘×’ denotes multiplication.

(26) $$P=\vec{R_{1}}<TDR,$$

(27) IDX=(P==0),

(28) $$Z=R_{2}\Theta \;IDX+\vec{R_{3}}\Theta \;\left(∼IDX\right),$$

(29) $$TDR=1-\frac{iter}{max\;iter},$$

Let *P* be a binary vector composed of 0 and 1, both $\vec{R_{1}}$ and $\vec{R_{3}}$ are random vectors uniformly distributed within the range [0, 1]; $R_{2}$ is a scalar random number in the range [0, 1]. The variable IDX denotes the set of indices in the random vector $R_{1}$ that satisfies P=0. The symbol Θ represents the dot product operation. TDR is an adaptive mechanism that defines a coefficient linearly decreasing from 1 to 0, with iter denoting the current iteration number.

A random number *Rand* is generated. If *Rand* exceeds the crossover probability PC, the position of the foal is updated according to Equation (26); otherwise, the update is performed based on Equation (30).

(30) $$\begin{array}{c} X_{G,k}^{p}=Crossover\left(X_{G,i}^{q},X_{G,j}^{z}\right),i\neq j\neq k,p=q\leq end,\\ Crossove=Mean, \end{array}$$

The $X_{G,i}^{q}$ represents the position of individual *q* in group *i* after it returns to group *i* after outliers, and similarly, the $X_{G,j}^{z}$ represents the position of individual *z* in group *j* after it returns to group *j*. $X_{G,k}^{p}$ also represents the offspring individual produced by the mating of individual *q* in group *i* and individual *z* in group *j*. The positions of the two parents are represented inside the brackets in (30). The end denotes the last foal in the group. Crossover means that the *q*th foal from group *i* mates with the *z*th foal from group *j*, and the resulting offspring becomes the *p*th horse in group *k*. The value of the *p*th foal is obtained by calculating the average of the *q*th and *z*th foal.

The leader position is updated as described in Equation (31).

(31) $$\bar{Stallion_{Gi}}=\left\{\begin{array}{ccc} 2Zcos\left(2\pi RZ\right)\times \left(WH-Stallion_{Gi}\right)+WH, & \;\;\;\mathrm{if} & R>0.5,\\ 2Zcos\left(2\pi RZ\right)\times \left(WH-Stallion_{Gi}\right)-WH, & \;\;\;\mathrm{if} & R\leq 0.5, \end{array}\right.$$

where $\bar{Stallion_{Gi}}$ denotes the next position of the leader in group *i*, while WH represents the current global best solution. $Stallion_{Gi}$ is the current position of the group *i* leader, and *Z* is an adaptive parameter calculated by (32). *R* is a random number within [−2, 2], and π = 3.14.

The fitness function is represented by (32), where fitness is the individual’s fitness, and L(c) represents the path length:

(32) fitness=1/L(c),

The pseudo-code of WHO is shown in Algorithm 1.
**Algorithm 1** Pseudo-code of WHO**Require:** Objective function f(x)**Ensure:** The best solution $X_{best}$1:Initialize the wild horse population(popN), X, PS, PC, MI, NS=popN∗PS, Nf=popN∗(1−PS) //Initialize the population.2:Iter=13:Create groups and select leaders
4:Find the best horse as the solution // Find the global optimal value
5:**while** Iter<=MI **do**6:       Calculate fitness by Equation (32) // Calculate the population fitness
7:       Calculate TDR by Equation (29)
8:       **for** i=1:NS **do**
9:          Update the position of the stallion by Equation (31)
10:        **for** k=1:Nf/NS **do**
11:              Update the position of the foal by Equation (25)
12:        **end for**
13:        Select leaders // Find the optimal values for each group
14:        Find the best leader // Find the global optimal value
15:       **end for**
16:**end while**17:Return global best solution $X_{best}$


### 5.5. Hybrid Artificial Bee Colony–Wild Horse Optimizer (HABC-WHO)

To improve the accuracy of the solution, we propose a hybrid optimization algorithm named the Hybrid Artificial Bee Colony–Wild Horse Optimizer (HABC-WHO). This hybrid approach is designed to leverage the complementary strengths of both algorithms. The core idea of HABC-WHO is to enhance the global search capability of the Wild Horse Optimizer (WHO) by integrating the exploitation ability of the Artificial Bee Colony (ABC) algorithm [47]. Specifically, during the iteration process, the worst individual in the WHO population is replaced with the best solution obtained from the ABC component. This mechanism allows the algorithm to avoid premature convergence and maintain population diversity, thereby improving its convergence speed and solution accuracy.

The Artificial Bee Colony (ABC) algorithm, originally proposed by Karaboga from Erciyes University in Turkey [47], is a swarm-based optimization technique inspired by the intelligent foraging behavior of honeybee colonies. By embedding the ABC’s intelligent local search into the WHO framework, the proposed HABC-WHO aims to strike a better balance between exploration and exploitation, making it more effective in solving complex optimization problems.

The HABC-WHO algorithm procedure is shown in Algorithm 2.

The initial solutions are constructed using a clustering approach. All customers are clustered using k-means clustering, and assigned to the nearest satellite. The primary path is initialized by randomly arranging the customers to form the initial route.
**Algorithm 2** Pseudo-code of HABC-WHO**Require:** Objective function f(x)**Ensure:** The best solution $X_{best}$1:Initialize the wild horse population(popN), X, PS, PC, MI, NS=popN∗PS, Nf=popN∗(1−PS) //Initialize the population
2:Iter=1
3:Create groups and select leaders
4:Find the best horse as the solution // Find the global optimal value
5:**while** Iter<=MI **do**6:       Calculate fitness by Equation (32) // Calculate the population fitness
7:       Calculate TDR by Equation (29)
8:       **for** i=1:NS **do**
9:          Update the position of stallions by Equation (31)
10:          Large neighborhood operator //Apply large neighborhood operations for stallions
11:        **for** k=1:Nf/NS **do**
12:              Update the position of foals by Equation (25)
13:              Large neighborhood operator //Apply large neighborhood operations for stallions
14:        **end for**
15:        Find the bestfoal of each group
16:        **if**
bestfoal<leader **then**
17:              Exchange bestfoal, leader //The best foal in the group is exchanged with the stallion
18:        **end if**
19:        Find the best leader // Find the global optimal value
20:        $X_{best}$ decoding
21:       **end for**
22:       Assign the wild horse population to the ABC population
23:       Perform large neighborhood operation during the employed bee phase.
24:       Update the bee colony information.
25:       The best individual of ABC replaces the worst individual of WHO
26:       2-Opt operator
27:       Satellite subpath crossover operator
28:       **if**
$X_{best}$ < bestleader **then**
29:        Exchange bestleader, $X_{best}$
30:       **end if**
31:**end while**32: Return global best solution $X_{best}$


### 5.6. Search Strategy

To improve the convergence speed of the algorithm, we introduce different search strategies, including the large neighborhood search strategy, 2-opt strategy [48], and satellite subpath crossover strategy.

#### 5.6.1. Large Neighborhood Search

The basic Wild Horse Optimizer has a relatively slow convergence rate. To accelerate convergence, we introduce a large neighborhood search strategy [36], which includes destroy and repair operators. The destroy operator randomly removes several customers from a route and places them into a customer pool. The repair operator then reinserts the removed customers back into the satellite routes using a roulette wheel selection mechanism. This large neighborhood search strategy is applied separately to the routes in both the first and second stages.

#### 5.6.2. Two-Optimization (2-Opt) Operation

**Figure 4:**
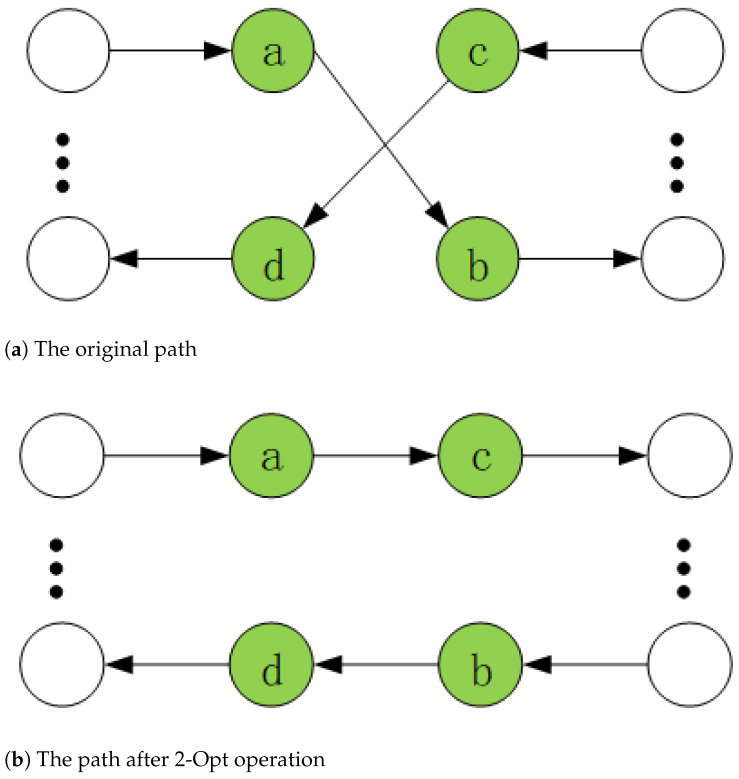
2-Opt operation.

To enhance local search capabilities, we incorporate the two-optimization (2-Opt) [48] search strategy. This strategy is applied to each satellite route by disconnecting two edges within a route and reconnecting them in a different configuration, as illustrated in Figure 4. If the new configuration yields a lower cost than the original, the new connection is adopted.

#### 5.6.3. Satellite Subpath Crossover Strategy

**Figure 5:**
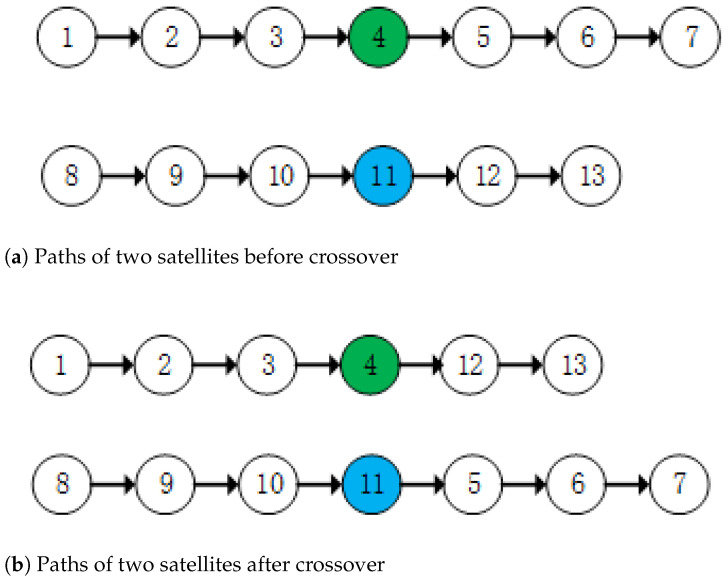
Satellite subpath crossover.

The subpaths between two satellites are crossed. A point is selected on each satellite’s subpath, dividing each subpath into two segments. Then, the two segments of each satellite’s subpath are exchanged. The satellite path crossover operation is shown in Figure 5. Figure 5a shows the paths of two satellites before crossover, and Figure 5b shows the paths of the two satellites after crossover.

## 6. Computational Tests

### 6.1. Parameter Settings for HABC-WHO

When the population size was set to popN = 50, the stallion proportion (PS) could be selected as either 0.2 or 0.5 to ensure an equal number of colts in each group. When PS=0.2, the number of foals in each group exceeded that of stallions, thereby promoting diversity among the colts within the group. Therefore, PS was set to 0.2 in these experiments. Extensive experimental evaluations revealed that the algorithm achieved optimal performance when the mating probability (*PC*) was set to around 0.13. Thus, PC was chosen as 0.13 in the experiments.

The following were the relevant parameter settings for HABC-DWHO:

- Population size: *popN* = 50.
- Stallion ratio: *PS* = 0.2.
- Mating probability: *PC* = 0.13.
- Maximum number of iterations: *MI* = 300.
- Number of stallions: *NS = popN × PS* = 10.
- Number of foals: *Nf = popN × (1 − PS)* = 40.

The computational configuration was as follows:

- OS: Windows 10 (×64).
- CPU: Intel Core i5-11400 (2.60 GHz).
- RAM: 16 GB.
- Language: Matlab 2016B.

The parameter settings for the three comparison algorithms were as follows: For the GA, the population size was set to 50, with a crossover probability of 0.8 and a mutation probability of 0.2. The maximum number of iterations was set to 300. For the DWHO, the population size was also set to 50, with a stallion ratio of 0.2 and a mating probability of 0.13, while the maximum number of iterations was kept at 300. For the DABC-FNS, the population size was set to 50, the limit parameter was set to 50, and the maximum number of iterations was set to 300.

### 6.2. Experimental Verification

Here, we used standard instances to verify the results of our algorithm. The results were compared with those obtained by a Genetic Algorithm (GA), a Discrete Wild Horse Optimizer (DWHO) [49], and a Discrete Artificial Bee Colony–Fixed Neighborhood Search (DABC-FNS) [42], as shown in Table 2, Table 3 and Table 4.

The Genetic Algorithm (GA) follows an evolutionary framework that balances exploration and exploitation strategies. Exploration is facilitated through crossover operators such as order crossover (OX) and partially mapped crossover (PMX), while exploitation is enhanced via mutation and local search techniques, including the 2-Opt heuristic and large neighborhood search (LNS). The DWHO is derived from the Wild Horse Optimizer (WHO) and incorporates three local search strategies—swap, reverse, and insertion operations—along with the largest-order-value (LOV) decoding technique [49]. The DABC-FNS framework is based on the discrete Artificial Bee Colony (ABC) algorithm with fixed neighborhood search strategies, including random single-point insertion, random sequence insertion, and random insertion. Moreover, a 2-opt strategy is applied within a fixed neighborhood to further refine local search performance [42].

The proposed algorithm was compared with the GA, DWHO, and DABC-FNS in solving the standard benchmark problems from sets 2, 3, and 4. As shown in Table 2, for the set 2 instances, the proposed algorithm significantly outperformed the GA and DWHO. In comparison with the DABC-FNS, the proposed method was superior in 10 instances, equivalent in 5 instances, and slightly worse in only 2 instances. In Table 3, the proposed algorithm again demonstrated superior performance compared to the GA and DWHO, with better results in 11 instances, equivalent performance in 4 instances, and slightly inferior results in 3 instances when compared to DABC-FNS. Furthermore, as indicated in Table 4, the proposed algorithm outperformed the other three algorithms in solving the set 4 instances, showcasing its superior solution capability. In Table 2 and Table 3, the negative sign indicates that the obtained solution was better than the best known solution.

**Figure 6:**
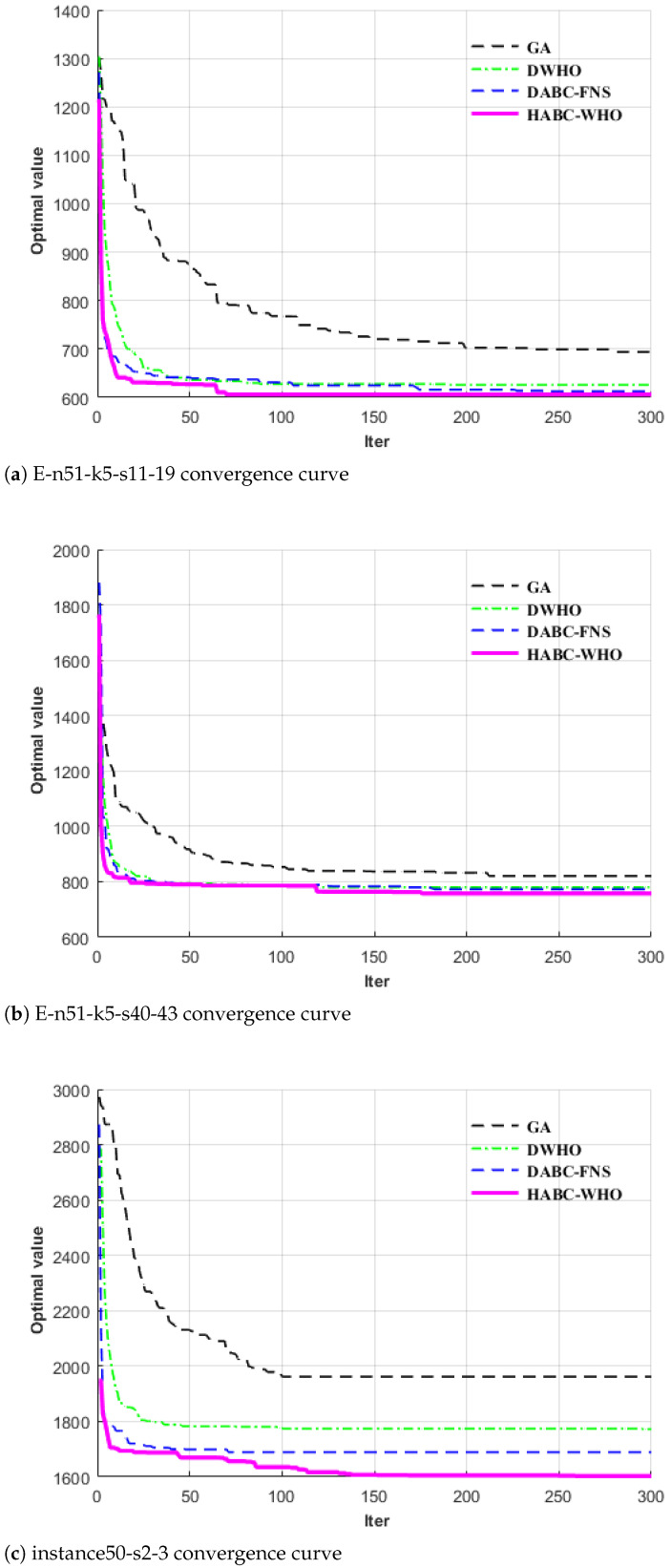
Comparison of convergence speeds of different algorithms.

To further verify the effectiveness of the algorithm proposed in this paper, we used the Wilcoxon’s rank sum test, a nonparametric statistic test, and the significance level α was set to 0.05. The symbols ‘+’, ‘=’, and ‘−’ denote that HABC-WHO was significantly better than, similar to, and significantly worse than its competitor on an instance, respectively, according to the *p* value. The comparison results are shown in Table 5, Table 6 and Table 7. It can be seen from Table 5, Table 6 and Table 7 that the solving ability of the algorithm proposed in this paper was significantly superior to that of the three algorithms being compared. From the convergence curves in Figure 6, it can be similarly observed that the proposed algorithm demonstrates superior solving capability compared to the three benchmark algorithms.

### 6.3. Time Complexity Analysis

The maximum number of iterations for the proposed algorithm is MI, and the population size of the HABC-WHO algorithm is *m*. The number of customers is *n*. The time complexity of population initialization is O(n), while both the herding and mating operations have a time complexity of O(n). The fitness evaluation requires O(n/2), the large neighborhood search operation has a complexity of O(n2), the subpath crossover operation is O(*n*/2), and the computation of the global best solution is O(*n*). Additionally, the Artificial Bee Colony (ABC) algorithm has a time complexity of $O(MI\cdot \left(n^{2}+m\cdot n\right))$. Therefore, the overall time complexity of the proposed algorithm is $O\left(MI\cdot m\cdot n^{2}\right)+O(MI\cdot \left(n^{2}+m\cdot n\right)$, which simplifies to $O(MI\cdot m\cdot n^{2})$.

## 7. Application to Real-World Problem

**Figure 7:**
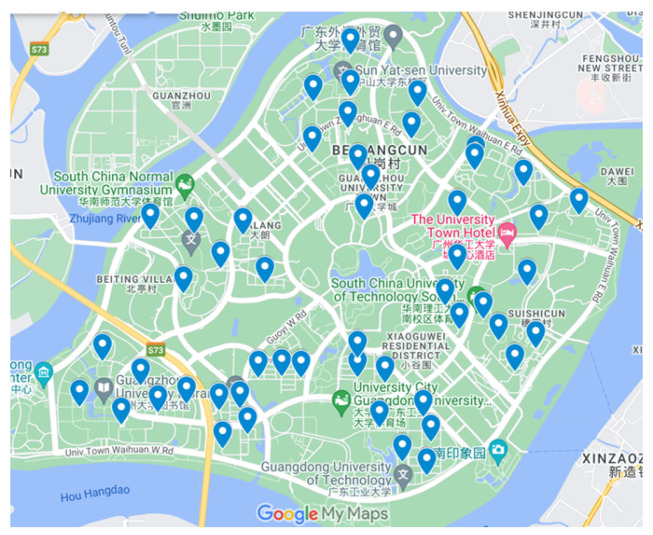
Distribution map of real-world problem.

In a university campus scenario under extreme weather conditions, it is essential to ensure continuous supply of daily necessities to students. Due to the extreme weather, traditional delivery methods and drone delivery are not feasible, so unmanned ground vehicles (UGVs) are employed. The case involved one distribution center, two satellites, and fifty terminal receiving users. The geographic distribution of the case is shown in Figure 7. For computational convenience, the Euclidean distance was used to measure distances between points. The HABC-WHO achieves an optimal solution of 51.85, demonstrating significant improvement over competing methods. Table 8 summarizes the comparative performance of the four algorithms on this instance. The results demonstrate that the proposed algorithm consistently outperforms the three competing methods across all comparison metrics.

## 8. Conclusions

Previous studies on two-echelon delivery problems primarily considered truck–drone or truck–unmanned vehicle combinations. This study, for the first time, introduced the two-echelon unmanned ground vehicle routing problem (2E-UGVRP), where unmanned ground vehicles (UGVs) were utilized in both delivery echelons. A corresponding mathematical model was formulated, and a novel solution approach, the hybrid Artificial Bee Colony–Wild Horse Optimizer (HABC-WHO), was proposed. This work addressed a research gap and provided new insights into unmanned logistics optimization. The HABC-WHO proposed in this paper demonstrated enhanced capability in solving the 2E-UGVRP problem. By incorporating strategies like large neighborhood search, 2-Opt local search, and satellite subpath crossover, the algorithm was shown to outperform others in solving 43 benchmark instances. Additionally, the application to a case study under extreme weather conditions revealed that the proposed algorithm’s global search strategy was significantly better than that of the compared algorithms, highlighting the effectiveness and superiority of the approach. Future research may focus on multi-objective extensions, such as incorporating time window constraints and customer satisfaction levels. In future work, we plan to explore the potential of this algorithm for solving other discrete combinatorial optimization problems, further expanding its applicability.

## Figures and Tables

**Table 1:** Review of two-echelon vehicle routing problem solution approaches.

Reference	Homogeneous Fleet	Objective	With Drone	Solution Approach	Year
Kergosien Y et al. [37]	Yes	Time	No	Genetic Algorithm, tabu search	2013
Murray C C & Chu A G [14]	Yes	Time	Yes	MILP, FSTSP	2015
Grangier P et al. [34]	Yes	Cost	No	Adaptive large neighborhood search	2016
X. Zhong et al. [19]	Yes	Cost	No	Artificial Bee Colony algorithm, Genetic Algorithm	2017
Carlsson J G, Song S [39]	Yes	Time	Yes	Horsefly algorithm	2018
Agatz N et al. [15]	Yes	Time	Yes	Integer program model, greedy partitioning heuristic	2018
Bouman P et al. [38]	yes	Cost	Yes	Dynamic programming	2018
Dellaert N et al. [27]	Yes	Cost	No	Branch-and-price algorithm	2018
Bouman P et al. [38]	Yes	Time	Yes	Dynamic programming approach, A* algorithm	2018
Yu K et al. [40]	Yes	Time	Yes	Generalized large neighborhood search solver, integer linear programming	2019
Schermer D et al. [41]	Yes	Time	Yes	VNS, tabu	2019
Breunig U et al. [20]	Yes	Cost	No	Large neighborhood search, exact mathematical programming algorithm	2019
Karak A.& Abdelghany, K [43]	Yes	Cost	Yes	Clarke and Wright algorithm	2019
Poikonen S et al. [44]	Yes	Time	Yes	Branch-and-bound	2019
Bevilaqua A et al. [13]	No	Cost	No	Lin–Kernighan heuristic	2019
Jie W et al. [21]	Yes	Cost	No	Combines a column generation and adaptive large neighborhood search	2019
Agárdi A et al. [22]	Yes	Cost	No	Hill climbing algorithm, Genetic Algorithm	2019
Marques G et al. [35]	Yes	Cost	No	Branch-cut-and-price	2020
Yu S et al. [2]	Yes	Cost	Yes	Mixed-integer program, hybrid metaheuristic	2020
Li H et al. [17]	Yes	Cost	Yes	Adaptive large neighborhood search heuristic	2020
Anderluh A et al. [36]	Yes	Cost	No	Large neighborhood search	2021
Mhamedi T et al. [29]	Yes	Cost	No	Branch-cut-and-price	2021
Dellaert N et al. [30]	Yes	Cost	No	Branch-and-price	2021
Zhou H et al. [31]	Yes	Cost	No	Variable neighborhood search, tabu search	2022
Marques G et al. [24]	Yes	Cost	No	Branch-cut-and-price	2022
Zhang L et al. [18]	No	Cost	No	Hybrid Genetic Algorithm	2023
Kim B et al. [32]	No	Cost	No	Adaptive large neighborhood search	2023
Zhou H et al. [16]	Yes	Time	Yes	Tabu search, exact branch-and-price algorithm	2023
Vincent F Y et al. [25]	Yes	Cost	No	Hybrid adaptive large neighborhood search	2023
Lehmann J & Winkenbach M [26]	Yes	Cost	No	Simulated annealing	2024

**Table 2:** Comparison results of set 2 benchmark instances.

Instance	BKS	GA	DWHO	DABC-FNS	HABC-WHO	
		Best	Best	Best	Best	Gap (%)
E-n22-k4-s06-17	417.07	424.81	424.81	417.07	417.07	0.00
E-n22-k4-s08-14	384.96	386.25	386.25	384.96	384.96	0.00
E-n22-k4-s09-19	470.60	476.13	476.13	476.13	470.72	0.03
E-n22-k4-s10-14	371.50	375.82	375.82	373.24	371.39	−0.03
E-n22-k4-s11-12	427.22	456.88	453.66	427.22	427.22	0.00
E-n22-k4-s12-16	392.78	425.49	423.55	392.78	392.78	0.00
E-n33-k4-s01-09	730.16	774.91	774.41	774.41	730.16	0.00
E-n33-k4-s02-13	714.63	848.45	745.27	736.33	714.64	0.00
E-n33-k4-s03-17	707.41	875.27	810.61	731.01	707.32	−0.01
E-n33-k4-s04-05	778.73	850.19	778.76	758.44	757.91	−2.67
E-n33-k4-s07-25	756.84	778.88	775.66	746.40	748.76	−1.07
E-n33-k4-s14-22	779.05	844.06	833.12	824.42	824.42	5.82
E-n51-k5-s02-04-17-46	530.76	594.71	668.87	571.58	570.31	7.45
E-n51-k5-s02-17	597.49	650.52	634.17	597.49	602.72	0.88
E-n51-k5-s06-12	554.80	607.4	590.89	567.88	567.18	2.23
E-n51-k5-s11-19	581.64	693.74	626.01	612.66	606.30	4.24
E-n51-k5-s27-47	538.22	612.35	600.82	564.15	563.92	4.77

**Table 3:** Comparison results of set 3 benchmark instances.

Instance	BKS	GA	DWHO	DABC-FNS	HABC-WHO	
		Best	Best	Best	Best	Gap (%)
E-n22-k4-s13-14	526.15	541.36	541.36	526.15	526.15	0.00
E-n22-k4-s13-16	521.09	546.23	546.23	521.77	518.69	−0.46
E-n22-k4-s13-17	496.38	496.38	496.38	496.38	496.38	0.00
E-n22-k4-s14-19	498.80	541.45	506.99	498.80	498.59	−0.04
E-n22-k4-s17-19	512.80	607.42	577.74	514.53	514.08	0.25
E-n22-k4-s19-21	520.42	550.67	527.48	520.42	520.42	0.00
E-n33-k4-s16-22	634.26	817.25	788.60	671.55	666.78	5.13
E-n33-k4-s16-24	666.02	826.11	781.40	668.81	666.35	0.05
E-n33-k4-s19-26	680.36	699.35	691.73	680.46	680.49	0.02
E-n33-k4-s22-26	680.37	767.73	761.86	676.63	680.89	0.08
E-n33-k4-s24-28	670.43	762.31	756.02	670.86	670.86	0.06
E-n33-k4-s25-28	650.58	771.73	712.44	664.55	651.40	0.13
E-n51-k5-s12-18	690.59	828.23	698.85	694.23	692.51	0.28
E-n51-k5-s12-41	683.05	883.95	715.01	695.58	693.85	1.58
E-n51-k5-s12-43	710.41	926.04	757.58	747.49	745.88	4.99
E-n51-k5-s39-41	728.54	789.74	752.16	759.53	746.71	2.49
E-n51-k5-s40-41	723.75	852.68	729.94	729.94	730.19	0.89
E-n51-k5-s40-43	752.15	820.71	779.74	772.75	757.45	0.70

**Table 4:** Comparison results of set 4 benchmark instances.

Instance	BKS	GA	DWHO	DABC-FNS	HABC-WHO	
		Best	Best	Best	Best	Gap (%)
Instance50-s2-01	1590.00	1987.97	1776.11	1626.16	1590.04	0.00
Instance50-s2-02	1442.00	1780.62	1497.63	1516.43	1470.23	1.96
Instance50-s2-03	1603.00	1961.93	1772.37	1689.15	1602.97	0.00
Instance50-s2-04	1440.77	1530.69	1484.05	1497.91	1441.78	0.07
Instance50-s2-05	2188.15	2280.75	2212.14	2226.00	2200.27	0.55
Instance50-s2-06	1310.80	1398.20	1313.32	1331.86	1311.12	0.02
Instance50-s2-07	1486.00	1857.16	1668.14	1616.66	1486.09	0.01
Instance50-s2-08	1369.78	1444.15	1371.50	1404.72	1369.78	0.00

**Table 5:** Wilcoxon test for the four algorithms for set 2.

Instance	GA	DWHO	DABC-FNS	HABC-WHO
E-n22-k4-s06-17	4.38$\times 10^{2}\pm$9.41$\times 10^{0}$+	4.25$\times 10^{2}\pm$2.33$\times 10^{-13}$+	4.17$\times 10^{2}\pm$1.17$\times 10^{-13}$=	4.17$\times 10^{2}\pm$1.17$\times 10^{-13}$
E-n22-k4-s08-14	4.04$\times 10^{2}\pm$1.43$\times 10^{1}$+	3.86$\times 10^{2}\pm$0.00$\times 10^{0}$+	3.85$\times 10^{2}\pm$0.00$\times 10^{0}$=	3.85$\times 10^{2}\pm$0.00$\times 10^{0}$
E-n22-k4-s09-19	5.00$\times 10^{2}\pm$1.70$\times 10^{1}$+	4.76$\times 10^{2}\pm$0.00$\times 10^{0}$+	4.76$\times 10^{2}\pm$1.30$\times 10^{-14}$+	4.73$\times 10^{2}\pm$2.41$\times 10^{0}$
E-n22-k4-s10-14	3.91$\times 10^{2}\pm$1.21$\times 10^{1}$+	3.76$\times 10^{2}\pm$1.17$\times 10^{-13}$+	3.74$\times 10^{2}\pm$1.96$\times 10^{0}$+	3.71$\times 10^{2}\pm$1.17$\times 10^{-13}$
E-n22-k4-s11-12	4.65$\times 10^{2}\pm$6.74$\times 10^{0}$+	4.54$\times 10^{2}\pm$0.00$\times 10^{0}$+	4.27$\times 10^{2}\pm$1.07$\times 10^{-4}$=	4.27$\times 10^{2}\pm$5.83$\times 10^{-14}$
E-n22-k4-s12-16	4.38$\times 10^{2}\pm$6.49$\times 10^{0}$+	4.24$\times 10^{2}\pm$5.83$\times 10^{-14}$+	3.93$\times 10^{2}\pm$4.76$\times 10^{-1}$+	3.93$\times 10^{2}\pm$1.55$\times 10^{0}$
E-n33-k4-s01-09	8.13$\times 10^{2}\pm$2.92$\times 10^{1}$+	7.74$\times 10^{2}\pm$0.00$\times 10^{0}$+	7.78$\times 10^{2}\pm$6.24$\times 10^{0}$+	7.34$\times 10^{2}\pm$1.62$\times 10^{0}$
E-n33-k4-s02-13	9.08$\times 10^{2}\pm$2.50$\times 10^{1}$+	7.45$\times 10^{2}\pm$2.33$\times 10^{-13}$+	7.55$\times 10^{2}\pm$7.56$\times 10^{0}$+	7.26$\times 10^{2}\pm$8.16$\times 10^{0}$
E-n33-k4-s03-17	9.39$\times 10^{2}\pm$2.81$\times 10^{1}$+	8.11$\times 10^{2}\pm$1.46$\times 10^{0}$+	7.43$\times 10^{2}\pm$8.15$\times 10^{0}$+	7.16$\times 10^{2}\pm$7.77$\times 10^{0}$
E-n33-k4-s04-05	9.37$\times 10^{2}\pm$3.66$\times 10^{1}$+	7.88$\times 10^{2}\pm$7.50$\times 10^{0}$+	7.62$\times 10^{2}\pm$4.97$\times 10^{0}$=	7.60$\times 10^{2}\pm$2.25$\times 10^{0}$
E-n33-k4-s07-25	8.35$\times 10^{2}\pm$2.33$\times 10^{1}$+	7.76$\times 10^{2}\pm$5.22$\times 10^{-14}$+	7.51$\times 10^{2}\pm$3.32$\times 10^{0}$=	7.51$\times 10^{2}\pm$2.98$\times 10^{0}$
E-n33-k4-s14-22	9.93$\times 10^{2}\pm$2.57$\times 10^{1}$+	8.33$\times 10^{2}\pm$3.50$\times 10^{-13}$+	8.28$\times 10^{2}\pm$4.17$\times 10^{0}$+	8.24$\times 10^{2}\pm$2.61$\times 10^{-14}$
E-n51-k5-s02-04-17-46	6.41$\times 10^{2}\pm$2.09$\times 10^{1}$+	6.69$\times 10^{2}\pm$3.50$\times 10^{-13}$+	5.78$\times 10^{2}\pm$3.63$\times 10^{0}$=	5.77$\times 10^{2}\pm$4.42$\times 10^{0}$
E-n51-k5-s02-17	7.12$\times 10^{2}\pm$3.56$\times 10^{1}$+	6.35$\times 10^{2}\pm$5.26$\times 10^{-1}$+	6.06$\times 10^{2}\pm$6.64$\times 10^{0}$-	6.08$\times 10^{2}\pm$4.59$\times 10^{0}$
E-n51-k5-s06-12	6.79$\times 10^{2}\pm$4.09$\times 10^{1}$+	5.93$\times 10^{2}\pm$2.34$\times 10^{0}$+	5.80$\times 10^{2}\pm$5.64$\times 10^{0}$=	5.78$\times 10^{2}\pm$5.65$\times 10^{0}$
E-n51-k5-s11-19	7.51$\times 10^{2}\pm$4.16$\times 10^{1}$+	6.27$\times 10^{2}\pm$1.28$\times 10^{0}$+	6.19$\times 10^{2}\pm$4.58$\times 10^{0}$+	6.13$\times 10^{2}\pm$3.46$\times 10^{0}$
E-n51-k5-s27-47	6.53$\times 10^{2}\pm$3.48$\times 10^{1}$+	6.04$\times 10^{2}\pm$2.57$\times 10^{0}$+	5.84$\times 10^{2}\pm$7.98$\times 10^{0}$=	5.82$\times 10^{2}\pm$6.67$\times 10^{0}$
+/=/−	17/0/0	17/0/0	10/6/1	

**Table 6:** Wilcoxon test for the four algorithms for set 3.

Instance	GA	DWHO	DABC-FNS	HABC-WHO
E-n22-k4-s13-14	5.64$\times 10^{2}\pm$2.06$\times 10^{1}$+	5.41$\times 10^{2}\pm$0.00$\times 10^{0}$+	5.26$\times 10^{2}\pm$1.17$\times 10^{-13}$=	5.26$\times 10^{2}\pm$1.17$\times 10^{-13}$
E-n22-k4-s13-16	5.54$\times 10^{2}\pm$4.27$\times 10^{0}$+	5.46$\times 10^{2}\pm$1.25$\times 10^{-13}$+	5.24$\times 10^{2}\pm$4.31$\times 10^{0}$+	5.19$\times 10^{2}\pm$2.33$\times 10^{-13}$
E-n22-k4-s13-17	5.06$\times 10^{2}\pm$1.04$\times 10^{1}$+	4.96$\times 10^{2}\pm$5.83$\times 10^{-14}$+	4.96$\times 10^{2}\pm$2.23$\times 10^{-3}$+	4.91$\times 10^{2}\pm$1.17$\times 10^{-13}$
E-n22-k4-s14-19	5.75$\times 10^{2}\pm$3.28$\times 10^{1}$+	5.07$\times 10^{2}\pm$0.00$\times 10^{0}$+	5.02$\times 10^{2}\pm$3.74$\times 10^{0}$=	5.01$\times 10^{2}\pm$2.25$\times 10^{0}$
E-n22-k4-s17-19	6.27$\times 10^{2}\pm$1.93$\times 10^{1}$+	5.78$\times 10^{2}\pm$0.00$\times 10^{0}$+	5.18$\times 10^{2}\pm$3.15$\times 10^{0}$-	5.18$\times 10^{2}\pm$3.96$\times 10^{0}$
E-n22-k4-s19-21	5.83$\times 10^{2}\pm$2.74$\times 10^{1}$+	5.27$\times 10^{2}\pm$1.17$\times 10^{-13}$+	5.29$\times 10^{2}\pm$9.14$\times 10^{0}$+	5.20$\times 10^{2}\pm$0.00$\times 10^{0}$
E-n33-k4-s16-22	8.53$\times 10^{2}\pm$2.65$\times 10^{1}$+	7.90$\times 10^{2}\pm$8.75$\times 10^{-1}$+	6.80$\times 10^{2}\pm$7.84$\times 10^{0}$+	6.67$\times 10^{2}\pm$1.09$\times 10^{-2}$
E-n33-k4-s16-24	8.47$\times 10^{2}\pm$1.92$\times 10^{1}$+	7.86$\times 10^{2}\pm$3.57$\times 10^{0}$+	6.74$\times 10^{2}\pm$7.60$\times 10^{0}$+	6.69$\times 10^{2}\pm$3.63$\times 10^{0}$
E-n33-k4-s19-26	7.24$\times 10^{2}\pm$1.46$\times 10^{1}$+	6.92$\times 10^{2}\pm$2.33$\times 10^{-13}$+	6.83$\times 10^{2}\pm$3.59$\times 10^{0}$=	6.82$\times 10^{2}\pm$1.98$\times 10^{0}$
E-n33-k4-s22-26	7.91$\times 10^{2}\pm$1.66$\times 10^{1}$+	7.62$\times 10^{2}\pm$2.33$\times 10^{-13}$+	6.80$\times 10^{2}\pm$3.73$\times 10^{0}$+	6.78$\times 10^{2}\pm$7.53$\times 10^{-1}$
E-n33-k4-s24-28	7.77$\times 10^{2}\pm$1.05$\times 10^{1}$+	7.57$\times 10^{2}\pm$5.59$\times 10^{-1}$+	6.79$\times 10^{2}\pm$7.56$\times 10^{0}$=	6.75$\times 10^{2}\pm$3.02$\times 10^{0}$
E-n33-k4-s25-28	8.19$\times 10^{2}\pm$2.51$\times 10^{1}$+	7.13$\times 10^{2}\pm$8.41$\times 10^{-1}$+	6.70$\times 10^{2}\pm$4.55$\times 10^{0}$+	6.56$\times 10^{2}\pm$4.11$\times 10^{0}$
E-n51-k5-s12-18	8.92$\times 10^{2}\pm$5.37$\times 10^{1}$+	7.01$\times 10^{2}\pm$3.24$\times 10^{0}$+	7.00$\times 10^{2}\pm$4.51$\times 10^{0}$+	6.98$\times 10^{2}\pm$2.91$\times 10^{0}$
E-n51-k5-s12-41	9.19$\times 10^{2}\pm$3.00$\times 10^{1}$+	7.23$\times 10^{2}\pm$4.00$\times 10^{0}$+	7.01$\times 10^{2}\pm$4.95$\times 10^{0}$-	7.02$\times 10^{2}\pm$5.28$\times 10^{0}$
E-n51-k5-s12-43	1.01$\times 10^{3}\pm$4.44$\times 10^{1}$+	7.64$\times 10^{2}\pm$2.18$\times 10^{0}$+	7.56$\times 10^{2}\pm$5.47$\times 10^{0}$-	7.57$\times 10^{2}\pm$5.74$\times 10^{0}$
E-n51-k5-s39-41	9.88$\times 10^{2}\pm$7.88$\times 10^{1}$+	7.56$\times 10^{2}\pm$2.22$\times 10^{0}$+	7.67$\times 10^{2}\pm$8.00$\times 10^{0}$+	7.53$\times 10^{2}\pm$2.76$\times 10^{0}$
E-n51-k5-s40-41	9.36$\times 10^{2}\pm$3.77$\times 10^{1}$+	7.42$\times 10^{2}\pm$6.70$\times 10^{0}$+	7.38$\times 10^{2}\pm$8.07$\times 10^{0}$+	7.31$\times 10^{2}\pm$4.55$\times 10^{0}$
E-n51-k5-s40-43	8.81$\times 10^{2}\pm$3.47$\times 10^{1}$+	7.89$\times 10^{2}\pm$2.43$\times 10^{0}$+	7.84$\times 10^{2}\pm$9.01$\times 10^{0}$+	7.65$\times 10^{2}\pm$5.83$\times 10^{0}$
+/=/−	18/0/0	18/0/0	11/4/3	

**Table 7:** Wilcoxon test for the four algorithms for set 4.

Instance	GA	DWHO	DABC-FNS	HABC-WHO
Instance50-s2-01	2.09 × 10^3^ ± 6.15 × 10^1^+	1.78 × 10^3^ ± 4.22 × 10^0^+	1.64 × 10^3^ ± 9.33 × 10^0^+	1.59 × 10^3^ ± 5.12 × 10^0^
Instance50-s2-02	1.91 × 10^3^ ± 7.47 × 10^1^+	1.50 × 10^3^ ± 2.22 × 10^0^+	1.52 × 10^3^ ± 8.69 × 10^0^+	1.48 × 10^3^ ± 6.92 × 10^0^
Instance50-s2-03	2.08 × 10^3^ ± 5.52 × 10^1^+	1.77 × 10^3^ ± 4.76 × 10^0^+	1.70 × 10^3^ ± 6.68 × 10^0^+	1.61 × 10^3^ ± 2.43 × 10^0^
Instance50-s2-04	1.64 × 10^3^ ± 5.37 × 10^1^+	1.49 × 10^3^ ± 5.11 × 10^0^+	1.50 × 10^3^ ± 5.82 × 10^0^+	1.45 × 10^3^ ± 4.86 × 10^0^
Instance50-s2-05	2.41 × 10^3^ ± 6.90 × 10^1^+	2.22 × 10^3^ ± 5.33 × 10^0^=	2.23 × 10^3^ ± 7.04 × 10^0^+	2.21 × 10^3^ ± 6.32 × 10^0^
Instance50-s2-06	1.55 × 10^3^ ± 7.92 × 10^1^+	1.32 × 10^3^ ± 9.77 × 10^0^+	1.34 × 10^3^ ± 8.63 × 10^0^+	1.31 × 10^3^ ± 3.89 × 10^0^
Instance50-s2-07	1.99 × 10^3^ ± 7.04 × 10^1^+	1.67 × 10^3^ ± 4.05 × 10^0^+	1.62 × 10^3^ ± 8.06 × 10^0^+	1.49 × 10^3^ ± 3.48 × 10^0^
Instance50-s2-08	1.55 × 10^3^ ± 5.46 × 10^1^+	1.37 × 10^3^ ± 1.27 × 10^0^+	1.41 × 10^3^ ± 8.97 × 10^0^+	1.37 × 10^3^ ± 1.27 × 10^0^
+/=/−	8/0/0	7/1/0	8/0/0	

**Table 8:** Comparison of solution results from different algorithms.

GA	DWHO	DABC-FNS	HABC-WHO
62.36	52.89	52.89	51.85

## Data Availability

The data presented in this study are available on request from the corresponding author.
